# Differentiating anomalous disease intensity with confounding variables in space

**DOI:** 10.1186/s12942-020-00231-3

**Published:** 2020-09-14

**Authors:** Chih-Chieh Wu, Sanjay Shete

**Affiliations:** 1 Department of Environmental and Occupational Health, College of Medicine, National Cheng Kung University, 1 University Road, Tainan, 701 Taiwan; 2 Department of Biostatistics, The University of Texas MD Anderson Cancer Center, Houston, TX USA

**Keywords:** Geographical disease cluster, Hierarchical, Incidence clustering, Sudden infant death Syndrome

## Abstract

**Background:**

The investigation of perceived geographical disease clusters serves as a preliminary step that expedites subsequent etiological studies and analysis of epidemicity. With the identification of disease clusters of statistical significance, to determine whether or not the detected disease clusters can be explained by known or suspected risk factors is a logical next step. The models allowing for confounding variables permit the investigators to determine if some risk factors can explain the occurrence of geographical clustering of disease incidence and to investigate other hidden spatially related risk factors if there still exist geographical disease clusters, after adjusting for risk factors.

**Methods:**

We propose to develop statistical methods for differentiating incidence intensity of geographical disease clusters of peak incidence and low incidence in a hierarchical manner, adjusted for confounding variables. The methods prioritize the areas with the highest or lowest incidence anomalies and are designed to recognize hierarchical (in intensity) disease clusters of respectively high-risk areas and low-risk areas within close geographic proximity on a map, with the adjustment for known or suspected risk factors. The data on spatial occurrence of sudden infant death syndrome with a confounding variable of race in North Carolina counties were analyzed, using the proposed methods.

**Results:**

The proposed Poisson model appears better than the one based on SMR, particularly at facilitating discrimination between the 13 counties with no cases. Our study showed that the difference in racial distribution of live births explained, to a large extent, the 3 previously identified hierarchical high-intensity clusters, and a small region of 4 mutually adjacent counties with the higher race-adjusted rates, which was hidden previously, emerged in the southwest, indicating that unobserved spatially related risk factors may cause the elevated risk. We also showed that a large geographical cluster with the low race-adjusted rates, which was hidden previously, emerged in the mid-east.

**Conclusion:**

With the information on hierarchy in adjusted intensity levels, epidemiologists and public health officials can better prioritize the regions with the highest rates for thorough etiologic studies, seeking hidden spatially related risk factors and precisely moving resources to areas with genuine highest abnormalities.

## Introduction

An important issue in spatial and temporal statistics is whether a set of discrete points are distributed randomly or they show a variety of signs of clustering. One of its major applications is in epidemiology; in particular, detecting and, more importantly, characterizing spatial and temporal clusters of adverse health events using existing health data collected on a basis of geographic units such as counties. The investigation of perceived incidence clusters or paucity of a certain disease is interesting in mathematics and probability per se. More importantly, we are interested in whether a finding indicating the presence of incidence anomalies (including clustering and paucity of disease incidence) will lead to a greater understanding of the etiology and underlying causal mechanism of disease or the identification of a common causal exposure for disease. With the identification of disease clusters of statistical significance, to determine whether or not the detected disease clusters can be explained by known or suspected risk factors is a logical next step.

The purpose of this paper is to develop and illustrate new statistical methods for differentiating incidence intensity of geographical disease clusters of peak incidence and low incidence, adjusted for covariates that are known or hypothesized risk factors, as well as testing for the presence of clustering. The methods are designed to recognize and construct hierarchical (in intensity) disease clusters of respectively high-risk areas and low-risk areas within close geographic proximity or contiguity on a map, including confounding variables as covariates. The hierarchy in covariate-adjusted intensity permits to occur between and within distinct geographical disease clusters. We propose to adjust for covariates by enumerating expected incidence of disease in each county through indirect standardization, conditioning on the total number of disease observed. The basic analysis is the one with no covariates. By including exactly one covariate at one time in further analyses, we can examine how the incorporated covariate affects the geographical disease incidence pattern. With information on geographical covariate-adjusted incidence clustering patterns on a map, we can determine whether or not the previously detected geographical disease clusters of peak incidence or paucity of incidence can be explained by the covariates incorporated. We are further interested if there still exist geographical disease clusters of incidence anomalies, after adjusting for known or hypothesized risk factors, which could lead to further investigation into other spatially related risk factors that are hidden otherwise.

The existing statistical methods for epidemiologic incidence anomaly patterns generally focus on detecting and characterizing large or peak incidence over a time, space, or space–time series. The statistical methods that we introduce in this paper focus on geographical incidence paucity of as well as peak incidence of adverse health events, including confounding variables as covariates. In epidemiology, testing for disease aggregations is used to identify the association between the possible risk factors and the incidence of disease. In contrast, the detection of an unusually low incidence of disease indicates the presence of protective factors or the absence of risk factors associated with the disease. We and others previously proposed and formulated statistical methods for detecting an unusually low incidence of disease in a unit of time in a discrete time series and evaluated their sensitivity, power, and applicability, using a temporal series of data on adolescent suicide from the US National Center for Health Statistics and on childhood Langerhans cell histiocytosis patients in Taiwan [1, 2]. We articulated that statistical methods that are sensitive to incidence paucity in a unit of time characterize opposite aspects of an observed incidence pattern and can be as meaningful and useful in epidemiology as the methods that focus on incidence clustering in our articles. The same rationales hold true for statistical methods for spatial epidemiology and spatial statistics, as proposed here.

We illustrate proposed statistical methods, using the data on the spatial occurrence of sudden infant death syndrome (SIDS) in North Carolina counties over a 4 year period, 1974–1978. One possible confounding variable for SIDS is race. Information on racial distribution of live births is available. This data set has been analyzed in a variety of statistical and epidemiologic reports [3–9]. We choose this data set for orientation in order to readily illustrate the difference in utilities and applicability among these spatial statistical methods. Atkinson provides an early review of the SIDS incidence and notes the statistically non-significant clustering of incidence in time within county by single years of calendar time, using the Ederer-Myers-Mantel test [3]. Cressie and his colleagues develop methods to model the spatial trend (large-scale variation) and spatial interdependence and autocorrelation (small-scale variation) in exploratory and confirmatory incidence analyses of this data [4–6]. Specifically, Cressie and Chan (1989) perform both unweighted and weighted linear regression and logistic regression of Freeman-Tukey transformed SIDS incidence on 5 explanatory variables, including population density, percentage urban, number of hospital beds, median family income, and Freeman-Tukey transformed non-white live birth rates, in modeling large-scale variation. They use the Markov random field in modeling small-scale variation [4]. Instead of classifying the counties at high risk or medium risk to SIDS, according to the magnitude and ranking of the observed rate of incidence alone, Symons, Grimson, and Yuan propose to determine the classification of the incidence risk level, accounting for the variance of the estimate of the rate in a Poisson process [9]. They employ a mixture of Poisson distributions to model the disease incidence and determine criteria for classification, using maximum likelihood and Bayesian methods. These authors all focus on relevant but different respects of the spatial statistics problems from what we propose here.

Both the map-based pattern recognition procedure [7] and the spatial scan statistic [8] can be used to identify disease clustering or disease clusters in a spatial point process in general. But they are designed for evaluating and characterizing different respects of spatially characteristic incidence clustering patterns and provide different information on spatial clustering. The spatial scan statistic is widely used for spatial cluster detection analysis and has been extended to a variety of models for detecting spatial, temporal, and space–time clusters, retrospectively or prospectively, using ordinal, survival-time, multi-nominal, normal, and longitudinal data etc.. The spatial scan statistic searches for spatial disease clusters not explained by a baseline spatial point process without specifying their size or location a priori. It is able to identify the approximate location and range of the most likely disease clusters and secondary disease clusters and to perform a significance test for each cluster, based on the maximum likelihood ratio and using Monte Carlo hypothesis testing. While the identified most likely disease clusters may not be the areas with the highest incidence by the spatial scan statistic. In contrast, the pattern recognition procedure prioritizes the areas with the highest rates and is designed to determine hierarchical incidence intensity levels of mutually adjacent areas with the highest rates geographically. It is noted that this procedure exclusively focuses on peak incidence and does not allow for covariates in determining the hierarchical intensity levels. We previously used the pattern recognition procedure to investigate the spatial clustering patterns of dengue outbreaks in Taiwan [10].

Our proposed methods generalize the pattern recognition procedure in several respects and have following features to address important problems:

1. We introduce the method for differentiating intensity of geographical disease clusters of low incidence in a hierarchical manner as well as testing for the presence of clustering.
2. We propose the methods for taking into account covariates that are known or hypothesized risk factors of disease in constructing hierarchical (in adjusted intensity) clusters of high-risk areas and low-risk areas close within geographic proximity, respectively.
3. The use of indirectly covariate-adjusted expected incidence permits the incorporation of covariates without the need of information on covariate-specific numbers of incidence in each area under study.
4. Two distinct probability models are proposed to assess the deviation between the observed incidence and covariate-adjusted expected incidence in each area.
5. Two distinct neighborhood systems, adjacency-based and distance-based in the definition of close geographical proximity, are used for proposed models.

Instead of dividing the counties into high- and medium-risk categories on the basis of the incidence rates used in the practice and in the existing literature, we propose to divide the counties into high-, medium-, and low-risk categories, then proceed to further differentiate incidence level of counties close within geographic proximity in the high- and low-risk categories respectively with and without the adjustment for confounding variables in a hierarchical manner.

The statistical methods that we propose in this report are not limited to advancing and generating studies of etiology of disease of interest with unknown causes. With the information on hierarchy in adjusted intensity levels, epidemiologists and public health officials can better prioritize the regions with the highest rates for thorough etiologic studies, seeking hidden spatially related risk factors, and precisely moving resources to areas with genuine highest abnormalities.

## Methods

In this section, we first introduce the method for differentiating intensity of geographical disease clusters of low incidence as well as testing for the presence of clustering. It is based on the extension of the existing pattern recognition procedure, which focuses on hierarchical clusters of high incidence [7]. Next, we generalize these methods by taking into account covariates that are known or hypothesized risk factors of the disease. We consider a covariate of race for SIDS in this application. Thirdly, we consider two distinct neighborhood systems for the North Carolina counties in the application of the proposed spatial statistical models, which are adjacency-based and distance-based in the definition of geographical proximity. We illustrate proposed statistical methods, using the data on the spatial occurrence of SIDS in North Carolina counties in 1974–1978.

### Study population

The data on SIDS patients with a confounding variable of race in 100 North Carolina counties provide an opportunity to illustrate the applications of the methods that we propose for geographical disease anomalies. SIDS is the third leading cause of all infant mortality in the US and remains the leading cause of death in infants aged from 1 month to 1 year. Its exact cause remains unknown. The frequency of SIDS appears to be influenced by social, economic, and cultural factors, such as maternal education, race or ethnicity, and poverty. Racial disparity in infants who died of SIDS has persisted. The rate of SIDS in non-Hispanic African American infants and American Indian/Alaskan Native infants remains more than twice that of non-Hispanic white infants in 2016 [11].

The data on the spatial occurrence of SIDS in North Carolina counties over a 4 year period, from July 01, 1974 to June 30, 1978, is used for illustration of our proposed methods. The information contained in this data set include the number of SIDS and the number of live births during this period for each of the 100 counties of North Carolina, as well as the county-seat locations. The number of live births was stratified into whites and non-whites for each of the 100 counties. The total number of live births was 329,962, in which the numbers of white and non-white births were 224,881 and 105,081, respectively. The total number of SIDS was 667, in which the numbers of white and non-white SIDS were 268 and 399, respectively. The state-wide incidence rate was 2.021 in deaths per 1000 live births. The overall incidence rates for the entire state by race were 1.192 for white children and 3.797 for non-white children per 1000 live births. Non-white SIDS rate was more than 3 times higher than that of whites, with the result that although non-whites accounted for only 31.85% of the live births in the state during the study period, they accounted for 59.82% of all the SIDS cases reported. Details of the data sources and data collection methods have been described elsewhere [4].

**Fig. 1:**
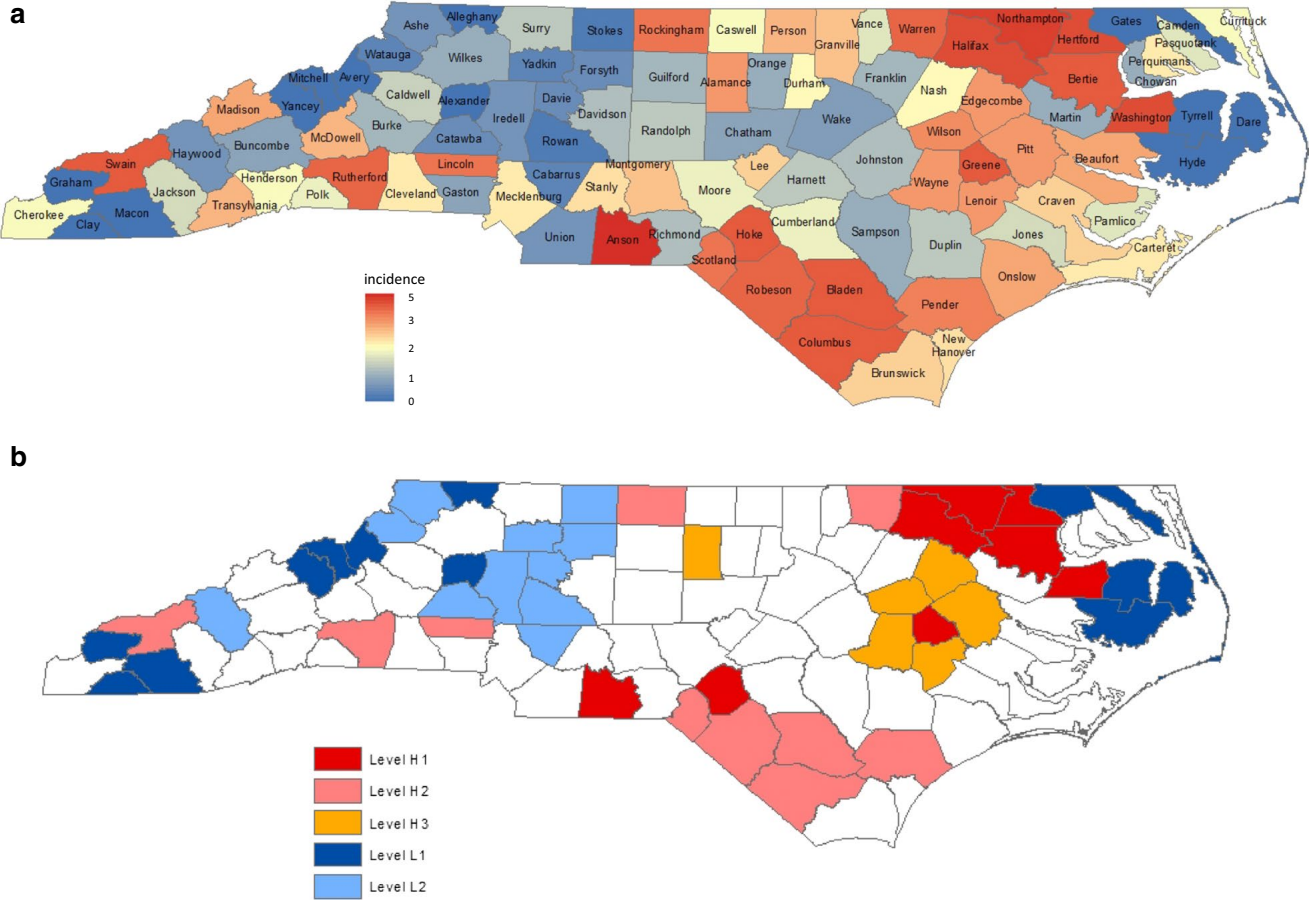
**a** County-specific SIDS incidence intensity map in North Carolina with county names. **b** County-specific SIDS incidence intensity-level map in North Carolina

Two distinct neighborhood systems for the North Carolina counties systems, distance-based and adjacency-based in the definition of close geographical proximity, are used for proposed models. They were determined by the criteria of being within 30 miles between the seats of 2 counties [4] and of sharing common geographical boundaries between 2 counties [5], respectively. The map on the 100 counties of North Carolina with county names is presented in Fig. 1a.

### Existing map-based pattern recognition procedure

The method developed by Mantel [12] was generalized by Cliff and Ord, who proposed the test statistic ***B***_***1***_** = (1/2) Σ ω**^***1***^_***ij***_*** x***_***i***_*** x***_***j***_ where ***x***_***i***_** = 1** if area ***i*** is a high-risk area for some disease and ***0*** otherwise, and where ***ω***^***1***^_***ij***_** = 1 **if areas ***i ***and* j* are mutually adjacent geographically and ***0*** otherwise, ***ω***^***1***^_***ij***_** = ω**^***1***^_***ji***_,* ω*^***1***^_***ii***_** = 0** [13]. The sum ranges over all pairs of areas. It is an adjacency-based test statistic that measures spatial autocorrelation for binary data and uses the distribution of the number of adjacencies of geographic units. When high-risk areas tend to be geographically adjacent to each other, the value of ***B***_***1***_ tends to be large. Using the test statistic ***B***_***1***_, one can test the null hypothesis of the random allocation of high-risk areas over the geographical region; that is, high-risk areas do not cluster. Cliff and Ord derived the expressions for the mean and variance of ***B***_***1***_ under the assumptions of binomial and hypergeometric distributions [14]. We note that lower *p* values of ***B***_***1***_ indicate high degrees of clustering or tight clustering, which conform to the adjacency-based definition of a cluster rather than being interpreted in the usual sense in this context.

Instead of selecting a specific threshold rate of incidence, the procedure proposes to first list the areas under study in rank order based on the disease intensity rates. It starts with classifying the 2 top ranking areas as high-risk areas and calculates the value of ***B***_***1***_. The procedure proceeds, letting the threshold rate of incidence vary downwards continuously, in which case it includes exactly one area with high rate at one time, up to an upper limit, such that at most we include, say, 25% of the total areas under study. Thus, the procedure subsequently includes the area with the 3rd highest rate and the other 2 areas with higher rates as high-risk areas and calculates the corresponding value of ***B***_***1***_. The *p* value is the probability that ***B***_***1***_ is equal to or higher than the observed number of adjacencies involved between these 3 areas with the highest disease intensity rates. Therefore, the procedure provides the *p *value of ***B***_***1***_ when the *k* top ranking areas among all areas under study are classified as high-risk areas for each *k* where *k* = 2, 3, 4….. The main feature of the procedure is to determine the hierarchical incidence intensity pattern through the distribution of *p* values of ***B***_***1***_ for *k* = 2, 3, 4…., which is illustrated in an application to the SIDS data in North Carolina in 1974–1978 [7]. Figure 1a presents the county-specific SIDS incidence intensity map in this period.

Instead of relying on the assumptions associated with the asymptotically normal distribution [14] or using Monte Carlo method [7], we propose to use simulation-based permutations using 1 million replicates based on the exact county boundary map, defined by ***ω***^***1***^, to obtain the null distribution of ***B***_***1***_. The basic geographic unit used in this report is a county. The distribution was simulated by randomly selecting exactly *k* counties among the 100 counties of North Carolina 1 million times and counting the number of the adjacent pairs appearing among the *k* counties for each of the 1 million replicates. The information on sharing common boundaries among North Carolina counties that we use here is available in the literature [5]. This process was applied for *k* = 2, 3, 4… 25. Each of the 24 distributions of ***B***_***1***_ for *k* = 2, 3, 4… 25 is given in Table 1. We also used simulation-based permutations using 1 million replicates based on the exact district boundary map under study to obtain the null distribution of ***B***_***1***_ in a study of dengue fever in Taiwan previously [10].

**Table 1:** Frequency distributions of the number of adjacencies simulated on the basis of 1 million random selections in North Carolina counties (1985)

Test Statistic B																			
	0	1	2	3	4	5	6	7	8	9	10	11	12	13	14	15	16	17	18
Number of risk districts																			
2	950,159	49,841																	
3	857,308	137,832	3914	946															
4	732,054	242,499	21,366	3810	227	42	2												
5	590,814	336,495	59,444	11,445	1480	287	32	3											
6	450,382	396,717	118,785	27,420	5405	1073	180	31	7										
7	323,334	411,171	189,011	57,366	14,760	3364	784	170	30	9	1								
8	218,544	381,058	253,117	101,837	32,717	9521	2435	588	133	44	5	1							
9	138,529	321,211	291,730	155,654	62,603	21,215	6537	1836	521	125	30	6	1	2					
10	82,053	245,418	296,086	206,466	104,215	43,060	15,270	5185	1577	480	147	31	9	3					
11	45,477	171,729	266,959	238,850	150,703	75,643	32,117	12,086	4354	1410	461	155	36	16	4				
12	23,242	109,855	216,702	245,088	190,909	115,006	57,804	25,338	10,294	3810	1291	461	144	39	11	4	2		
13	11,276	64,768	157,128	222,148	213,151	155,271	92,672	47,274	21,719	8916	3664	1308	469	156	51	18	5	5	
14	4973	34,446	103,575	179,331	210,320	185,701	129,737	77,672	40,753	19,452	8312	3504	1423	543	181	47	26	4	
15	2051	17,019	61,391	129,783	184,715	193,134	161,433	112,320	68,674	36,986	18,171	8433	3616	1380	568	220	57	33	12
16	781	7534	32,853	83,879	143,272	180,165	176,656	144,634	100,421	62,336	34,721	17,760	8379	3751	1696	715	282	105	36
17	261	3105	16,147	48,582	99,058	148,213	172,523	163,592	131,300	92,051	58,418	33,108	17,578	8675	4175	1860	780	354	137
18	106	1178	6954	25,289	60,982	108,386	147,655	163,622	151,691	122,079	87,207	55,888	32,987	18,068	9481	4634	2096	966	435
19	20	412	2772	12,016	33,858	70,109	111,956	144,068	155,078	142,991	115,384	83,703	54,993	33,762	18,945	10,204	5244	2438	1172
20	5	126	1085	5004	16,238	39,617	74,740	112,616	139,725	147,194	136,048	111,012	82,284	55,470	35,209	20,686	11,453	5813	2989
21		32	315	1856	7269	20,186	44,377	77,318	110,293	133,710	140,236	130,029	108,697	82,489	56,797	36,886	22,490	12,829	7069
22		10	87	575	2726	9026	23,189	46,437	76,994	106,700	128,272	134,653	126,312	106,480	82,276	59,380	39,719	24,942	14,833
23		5	21	187	933	3459	10,503	24,608	46,971	74,885	102,107	122,616	128,487	122,631	106,326	84,294	62,080	43,120	27,884
24			1	40	313	1251	4274	11,475	25,062	45,890	71,740	97,160	116,139	123,762	119,692	105,415	86,590	65,465	46,989
25			1	16	79	365	1488	4687	11,584	24,737	44,243	67,097	91,804	110,020	119,206	116,598	105,837	88,762	69,279

Test Statistic B																			
	19	20	21	22	23	24	25	26	27	28	29	30	31	32	33	34	35	Mean	Variance
Number of risk districts																			
2																		0.049841	0.047357
3																		0.14850	0.139951
4																		0.297791	0.278328
5																		0.497286	0.462137
6																		0.744885	0.685536
7																		1.043376	0.95444
8																		1.391523	1.26439
9																		1.785891	1.604608
10																		2.235957	1.996241
11																		2.735061	2.42580
12																		3.278483	2.869409
13	1																	3.875225	3.367968
14																		4.522114	3.886849
15	2	1	1															5.219609	4.45820
16	16	7		1														5.969317	5.058543
17	49	23	9	2														6.7586	5.662858
18	196	60	23	8	5	2	1	1										7.603729	6.31320
19	492	223	88	50	12	9	1											8.49114	6.98076
20	1513	635	307	140	56	20	9	6										9.441983	7.673056
21	3690	1852	898	376	160	91	30	14	5	4	2							10.4340	8.406782
22	8401	4453	2352	1171	565	256	117	37	23	12	2							11.47922	9.146078
23	17,106	10,097	5607	3026	1526	813	389	163	86	36	24	4	4	1		1		12.57792	9.92431
24	31,385	19,974	12,273	7059	3861	2141	1007	550	277	119	53	21	12	7		3		13.71906	10.72648
25	50,689	35,307	23,681	14,750	8817	5026	2781	1558	785	420	202	116	39	13	11	1	1	14.90769	11.5430

### Hierarchical clusters of neighboring low-risk areas

In the existing map-based pattern recognition procedure, *k* indicates the number of top ranking counties of North Carolina with the higher intensity rates, classified as high-risk counties. While focusing on geographical disease clusters of incidence paucity, we propose to use *k* to indicate the number of top ranking counties with the lower intensity rates, classified as low-risk counties. Using the pattern recognition procedure, we can determine the hierarchical (in intensity) spatial clusters of incidence paucity correspondingly.

### Methods with adjustment of covariates

We propose to adjust for the effects of confounding variables by enumerating expected incidence (or number) of disease in each county through indirect standardization, conditioning on the total number of disease observed. We define a measure to be the expected number of patients with disease of interest in a spatial unit of the study region, a county of North Carolina in this application, with *μ(A*_*i*_*)* = expected number of patients in county *A*_*i*_. Assuming that the incidence for sub-population* j* equals *E*_*j*_ in the entire study region and the size of sub-population *j* in county *A*_*i*_ is *a*_*ij*_, the expected incidence in county *A*_*i*_ is equal to the summation of the products of *E*_*j*_ and *a*_*ij*_, across all *j*, *μ(A*_*i*_*)* = ${\sum }_{j}{a}_{ij}{E}_{j}$. This indirectly covariate-adjusted expected incidence for a county permits us to incorporate covariates with no need of information on covariate-specific numbers of incidence in every county. With the use of indirect standardization, the generalization to multiple covariates, adjusted for multiple confounding variables, is immediate.

The overall incidence rates for the state of North Carolina by race were 1.192 for white children and 3.797 for non-white children per 1000 live births in 1974–1978. With the information on the white and non-white numbers of live births for each of the 100 North Carolina counties [4], an expression of the race-adjusted expected incidence in county *A*_*i*_ is *μ(A*_*i*_*)* = 1.192 × white births + 3.797 × non-white births, which is proportional to the expected number of SIDS.

We propose two models for assessing the discrepancy between the observed incidence and the county-specific expected covariate-adjusted incidence: the standardized morbidity ratio (SMR) and a Poisson model. The SMR, the ratio of the observed number of incidence to the expected number of incidence, provides a direct quantitative measure of the overall discrepancies between the observed incidence and expected covariate-adjusted incidence per county.

Alternatively, we propose a Poisson probability model for assessing the probability of the deviation of a particular frequency to be attributed to sampling fluctuations. We propose to adjust for covariates under the assumption of Poisson random variables, which provide a crude way to account for the unequal variances of the county rates. The rank order is based on the probability associated with the assessment of the discrepancy between the observed incidence and county-specific expected covariate-adjusted incidence in a county. The Poisson distributional assumptions were previously employed by several investigators in studies of SIDS data [5, 6, 8, 9]. The existing literature generally assume a batch of independently and identically distributed Poisson random variables for distinct counties with the null hypothesis of homogeneity of state-wide incidence [5, 6, 9]. In this report, we assume county-specific Poisson probability models whose mean depends on the expected covariate-adjusted incidence for each county.

Using the same expression of the race-adjusted expected incidence *μ(A*_*i*_*)* for county *A*_*i*_, we calculate the probability of departure from expected race-adjusted incidence for each county, based on the assumption of Poisson distribution. It is defined as follows, *N*_*i*_ = the number of incidence in county *A*_*i*_,

$$D_{i} = \mathop \sum \limits_{{k \ge N_{i} }} exp\left( { - \mu \left( {A_{i} } \right)} \right)\mu \left( {A_{i} } \right)^{k} /k!\,\;{\text{for}}N_{i} \, \ge \,\mu \left( {A_{i} } \right);$$

$$D_{i} = \mathop \sum \limits_{{k \le N_{i} { }}} exp\left( { - \mu \left( {A_{i} } \right)} \right)\mu \left( {A_{i} } \right)^{k} /k!\;{\text{for N}}_{{\text{i}}} \, \le \,\mu \left( {{\text{A}}_{{\text{i}}} } \right).$$

A small value of this probability ***D***_***i***_ indicates that the SIDS incidence at county *A*_*i*_ is unusually high beyond the effect of race, if the observed incidence large than expected race-adjusted incidence, or that the SIDS incidence at county *A*_*i*_ is unusually low beyond the effect of race, if the observed incidence smaller than expected race-adjusted incidence.

The step-by-step guidelines for the use of the proposed SMR models are:

1. Compute covariate-adjusted expected incidence for each area under study.
2. List the areas with the highest (lowest) rates of SMR in rank order, up to an upper limit, say, 25 percent of the total areas under study.
3. Like the ordinary map-based pattern recognition procedure, start with classifying the 2 top ranking areas as high-risk (low-risk) areas and calculate the value of ***B***_***1***_.
4. Proceed successively, including exactly one area with the high (low) rate of SMR next according to the rank order and the other areas with higher (lower) rates as high-risk (low-risk) areas at each step with the use of ***B***_***1***_.
5. Determine the areas with the lowest *p* values of ***B***_***1***_ relative to surrounding *p* values in the ranking.
6. The hierarchical characterization is constructed, based on the use of the inclusion points of these areas determined in the previous step.

The step-by-step guidelines for the use of the proposed Poisson models are:

1. Compute covariate-adjusted expected incidence for each area under study.
2. Calculate the probability of departure from expected covariate-adjusted incidence for each area based on the assumption of Poisson distribution.
3. List the areas with the smallest probabilities in rank order and the observed incidence larger (smaller) than expected incidence, up to an upper limit, say, 25 percent of the total areas under study.
4. Like the ordinary map-based pattern recognition procedure, start with classifying the 2 top ranking areas as high-risk (low-risk) areas and calculate the value of ***B***_***1***_.
5. Proceed successively, including exactly one area with the small probability of Poisson distribution next according to the rank order and the other areas with smaller probabilities and the observed incidence larger (smaller) than expected incidence as high-risk (low-risk) areas at each step with the use of ***B***_***1***_.
6. Determine the areas with the lowest *p* values of ***B***_***1***_ relative to surrounding *p* values in the ranking.
7. The hierarchical characterization is constructed, based on the use of the inclusion points of these areas determined in the previous step.

## Results

In this section, we first present the analysis of high-risk areas and low-risk areas close within geographic proximity without adjustment of race, respectively. Secondly, we present the analysis with adjustment of race, using the proposed SMR model and Poisson model. Thirdly, in addition to an adjacency-based definition of geographical proximity, we consider and present the analysis based on the use of a distance-based neighborhood system.

### Analysis without adjustment for race

We first repeated the analysis, which was previously performed [7]. We present the cluster statistics for 25 counties with the higher rates in Table 2. Our results were slightly different from those presented in Table 3 of their article: (1) the test statistic ***B***_***1***_ in Table 2 was smaller than the corresponding one in Table 3 by 1 between Warren (12th in rank) and Lenoir (20th in rank) because the recognition of sharing common boundaries among North Carolina counties was different in certain counties by the authors; (2) the *p* values of ***B***_***1***_ in Table 2 were obtained by the simulation-based permutations using 1 million replicates shown in Table 1, rather than by the 2000 replicates of the Monte Carlo method in their article.

**Table 2:** Cluster statistics for counties with the higher rates

Rank order	County	Rate	B_1_	*P* value^1^	Risk level	B_2_	*P* value^2^
1	Anson	9.55	–		H1	–	
2	Northampton	6.33	0	1	H1	0	1
3	Washington	5.05	0	1	H1	0	$\text{9.97}{\times10}^{-1}$
4	Halifax	4.99	1	$\text{2.68}{\times10}^{-1}$	H1	1	$\text{2.20}{\times10}^{-1}$
5	Hertford	4.82	2	$\text{7.27}{\times10}^{-2}$	H1	2	$\text{4.87}{{\times10}}^{-2}$
6	Hoke	4.69	2	$\text{1.53}{\times10}^{-1}$	H1	2	$\text{1.06}{\times10}^{-1}$
7	Greene	4.60	2	$\text{2.65}{\times10}^{-1}$	H1	2	$\text{1.89}{\times10}^{-1}$
8	Bertie	4.53	6	$\text{3.21}{\times10}^{-3}$	H1	4	$\text{2.34}{\times10}^{-2}$
9	Bladen	4.49	6	$\text{9.06}{\times10}^{-3}$	H2	4	$\text{4.99}{\times10}^{-2}$
10	Columbus	4.48	7	$\text{7.43}{\times10}^{-3}$	H2	5	$\text{3.06}{\times10}^{-2}$
11	Swain	4.44	7	$\text{1.85}{\times10}^{-2}$	H2	5	$\text{6.13}{\times10}^{-2}$
12	Warren	4.13	8	$\text{1.61}{\times10}^{-2}$	H2	5	$\text{1.11}{\times10}^{-1}$
13	Rutherford	4.01	8	$\text{3.63}{\times10}^{-2}$	H2	5	$\text{1.85}{\times10}^{-1}$
14	Robeson	3.93	11	$\text{5.73}{\times10}^{-3}$	H2	8	$\text{2.62}{\times10}^{-2}$
15	Lincoln	3.61	11	$\text{1.43}{\times10}^{-2}$	H2	8	$\text{5.25}{\times10}^{-2}$
16	Rockingham	3.60	11	$\text{3.27}{\times10}^{-2}$	H2	8	$\text{9.45}{\times10}^{-2}$
17	Scotland	3.55	13	$\text{1.61}{\times10}^{-2}$	H2	9	$\text{8.30}{\times10}^{-2}$
18	Pender	3.26	15	$\text{8.43}{\times10}^{-3}$	H2	9	$\text{1.42}{\times10}^{-1}$
19	Wilson	2.97	16	$\text{9.73}{\times10}^{-3}$	H3	10	$\text{1.30}{\times10}^{-1}$
20	Lenoir	2.79	17	$\text{1.15}{\times10}^{-2}$	H3	11	$\text{1.22}{\times10}^{-1}$
21	Alamance	2.78	18	$\text{1.42}{\times10}^{-2}$	H3	11	$\text{1.97}{\times10}^{-1}$
22	Pitt	2.75	21	$\text{4.54}{\times10}^{-3}$	H3	13	$\text{1.16}{\times10}^{-1}$
23	Edgecombe	2.73	24	$\text{1.52}{\times10}^{-3}$	H3	16	$\text{3.80}{\times10}^{-2}$
24	Wayne	2.71	27	$\text{4.92}{\times10}^{-4}$	H3	19	$\text{1.19}{\times10}^{-2}$
25	Madison	2.61	27	$\text{1.59}{\times10}^{-3}$		19	$\text{2.47}{\times10}^{-2}$

**Table 3:** Cluster statistics for counties with the lower rates

Rank order	County	Rate	New birth	B_1_	*P* value^1^	Risk level	B_2_	*P* value^2^
100	Alexander	0	1333	–		L1	–	
99	Macon	0	797	0	1	L1	0	1
98	Avery	0	781	0	1	L1	0	1
97	Yancey	0	770	0	1	L1	1	$\text{2.20}{\times10}^{-1}$
96	Mitchell	0	671	2	$\text{7.27}{\times10}^{-2}$	L1	3	$\text{7.62}{\times10}^{-3}$
95	Dare	0	521	2	$\text{1.53}{\times10}^{-1}$	L1	3	$\text{2.04}{\times10}^{-2}$
94	Alleghany	0	487	2	$\text{2.65}{\times10}^{-1}$	L1	3	$\text{4.57}{\times10}^{-2}$
93	Gates	0	420	2	$\text{4.00}{\times10}^{-1}$	L1	3	$\text{9.06}{\times10}^{-2}$
92	Graham	0	415	3	$\text{2.49}{\times10}^{-1}$	L1	4	$\text{4.99}{\times10}^{-2}$
91	Hyde	0	338	4	$\text{1.70}{\times10}^{-1}$	L1	4	$\text{9.47}{\times10}^{-2}$
90	Camden	0	286	5	$\text{1.26}{\times10}^{-1}$	L1	4	$\text{1.62}{\times10}^{-1}$
89	Clay	0	284	6	$\text{9.92}{\times10}^{-2}$	L1	6	$\text{4.31}{\times10}^{-2}$
88	Tyrrell	0	248	7	$\text{8.36}{\times10}^{-2}$	L1	7	$\text{3.31}{\times10}^{-2}$
87	Stokes	0.62	1612	7	$\text{1.52}{\times10}^{-1}$	L2	7	$\text{6.33}{\times10}^{-2}$
86	Rowan	0.65	4606	7	$\text{2.50}{\times10}^{-1}$	L2	7	$\text{1.14}{\times10}^{-1}$
85	Cabarrus	0.73	4099	7	$\text{3.75}{\times10}^{-1}$	L2	8	$\text{9.45}{\times10}^{-2}$
84	Watauga	0.76	1323	8	$\text{3.49}{\times10}^{-1}$	L2	10	$\text{4.00}{\times10}^{-2}$
83	Yadkin	0.79	1269	8	$\text{4.86}{\times10}^{-1}$	L2	10	$\text{7.46}{\times10}^{-2}$
82	Davie	0.83	1207	10	$\text{3.27}{\times10}^{-1}$	L2	12	$\text{3.53}{\times10}^{-2}$
81	Forsyth	0.84	11,858	13	$\text{1.34}{\times10}^{-1}$	L2	15	$\text{8.03}{\times10}^{-3}$
80	Catawba	0.87	5754	14	$\text{1.43}{\times10}^{-1}$	L2	16	$\text{8.58}{\times10}^{-3}$
79	Ashe	0.92	1091	16	$\text{9.69}{\times10}^{-2}$	L2	18	$\text{4.53}{\times10}^{-3}$
78	Haywood	0.95	2110	16	$\text{1.72}{\times10}^{-1}$	L2	18	$\text{1.03}{\times10}^{-2}$
77	Iredell	0.97	4139	21	$\text{2.74}{\times10}^{-2}$	L2	23	$\text{6.70}{\times10}^{-4}$
76	Union	1.02	3915	22	$\text{3.45}{\times10}^{-2}$		24	$\text{8.03}{\times10}^{-4}$

The largest downward peaks in *p* values relative to surrounding *p* values occurred at the inclusion points of Bertie (8th in rank), Robeson (14th in rank), Pender (18th in rank), and Wayne (24th in rank) counties, shown in the *p* value^1^ column of Table 2. Robeson and Pender are close in location and on the rank scale. So the construction of the hierarchical characterization used the downward peaks at inclusion points of Bertie, Pender, and Wayne counties. Correspondingly, we determined the 3 groups of counties to use in constructing hierarchical clusters of mutually neighboring high-risk counties with 3 different levels of intensity. Level-H1 counties are the 8 top ranking counties; Level-H2, 10 counties ranking from 9 to 18; Level-H3, 6 counties ranking from 19 to 24. The overall incidence of the 8 Level-H1, 10 Level-H2, and 6 Level-H3 counties combined are 5.57, 3.95, and 2.79 per 1000 live births, respectively.

When the level-specific intensity is placed on the map, 3 hierarchical intensity clusters of high SIDS emerge and are respectively located in the northeast (6 counties: 5 Level-H1 and 1 Level-H2 counties) with incidence of 4.98, the south (6 counties: 1 Level-H1 and 5 Level-H2 counties) with incidence of 4.06, and the mid-east (6 counties: 1 Level-H1 and 5 Level-H3 counties) with incidence of 3.09 per 1000 live births, as shown in Fig. 1b. This hierarchical characterization is identical to the one in the previous study [7].

Previously, *k* indicates the number of top ranking counties of North Carolina with the higher intensity rates, classified as high-risk counties, given in Table 2. While focusing on geographical disease clusters of incidence paucity, *k* indicates the number of top ranking counties with the lower intensity rates, classified as low-risk counties. The 25 counties with the lowest rates were listed by rank according to their rates in Table 3. There were 13 counties with 0 SIDS but different numbers of live births. Because the larger the population base for the rate, the more stable and reliable the rate will be, we discriminated between these 13 counties according to the numbers of live births: the ones with larger numbers of live births were considered higher in ranking. The original test statistic ***B***_***1***_** = (1/2) Σ ω**^***1***^_***ij***_*** x***_***i***_*** x***_***j***_ was revised to be where ***x***_***i***_** = 1** if area ***i*** is a low-risk county for disease and ***0*** otherwise, and where ***ω***^***1***^_***ij***_** = 1 **if counties ***i ***and* j* are mutually adjacent geographically and ***0*** otherwise, ***ω***^***1***^_***ij***_** = ω**^***1***^_***ji***_,* ω*^***1***^_***ii***_** = 0**. It is noted that, with each *k*, the test statistic ***B***_***1***_ in Table 3 was smaller than the corresponding one in Table 2, indicating that the high-risk counties show higher degrees of clustering geographically than the low-risk counties.

Similarly, the method starts with classifying the 2 top ranking counties as low-risk counties and calculates the value of ***B***_***1***_. We proceed successively, including exactly one county with the low rate next according to the rank order and the other counties with lower rates as low-risk counties at each step with the use of ***B***_***1***_. We observed the large downward peaks in *p* values relative to surrounding *p* values at including Tyrrell (88^th^ in rank), Ashe (79th in rank), and Iredell (77th in rank) counties, shown in the *p* value^1^ column of Table 3. Ashe and Iredell are close on the rank scale. So the hierarchical characterization was determined using the downward peaks at inclusion points of Tyrrell and Iredell counties.

The use of the lowest *p* values at various counties in the rankings means to capture the groups of counties with highly tight clustering. The hierarchical (in intensity) spatial clusters of incidence paucity were optimally determined by using the inclusion points of Tyrrell and Iredell counties. We, therefore, determined the 2 groups of counties to use in constructing hierarchical clusters of mutually neighboring low-risk counties with 2 different levels of intensity. Level-L1 counties are the 13 top ranking counties with 0 SIDS; Level-L2, 11 counties ranking from 87 to 77. The overall incidence of the 13 Level-L1 and 11 Level-L2 counties combined are 0 and 0.81 per 1000 live births, respectively. The 3 hierarchical low-intensity clusters appear respectively in the northwestern region (6 counties: 4 Level-L1 and 2 Level-L2) with incidence of 0.28, the mid-western region (9 counties: 1 Level-L1 and 8 Level-L2) with incidence of 0.70, and the eastern coastal region (3 counties: 3 Level-L1) with incidence of 0 per 1000 live births. Figure 1b presents the county-specific SIDS incidence intensity-level map.

### Standardized morbidity ratio with adjustment for race

**Fig. 2:**
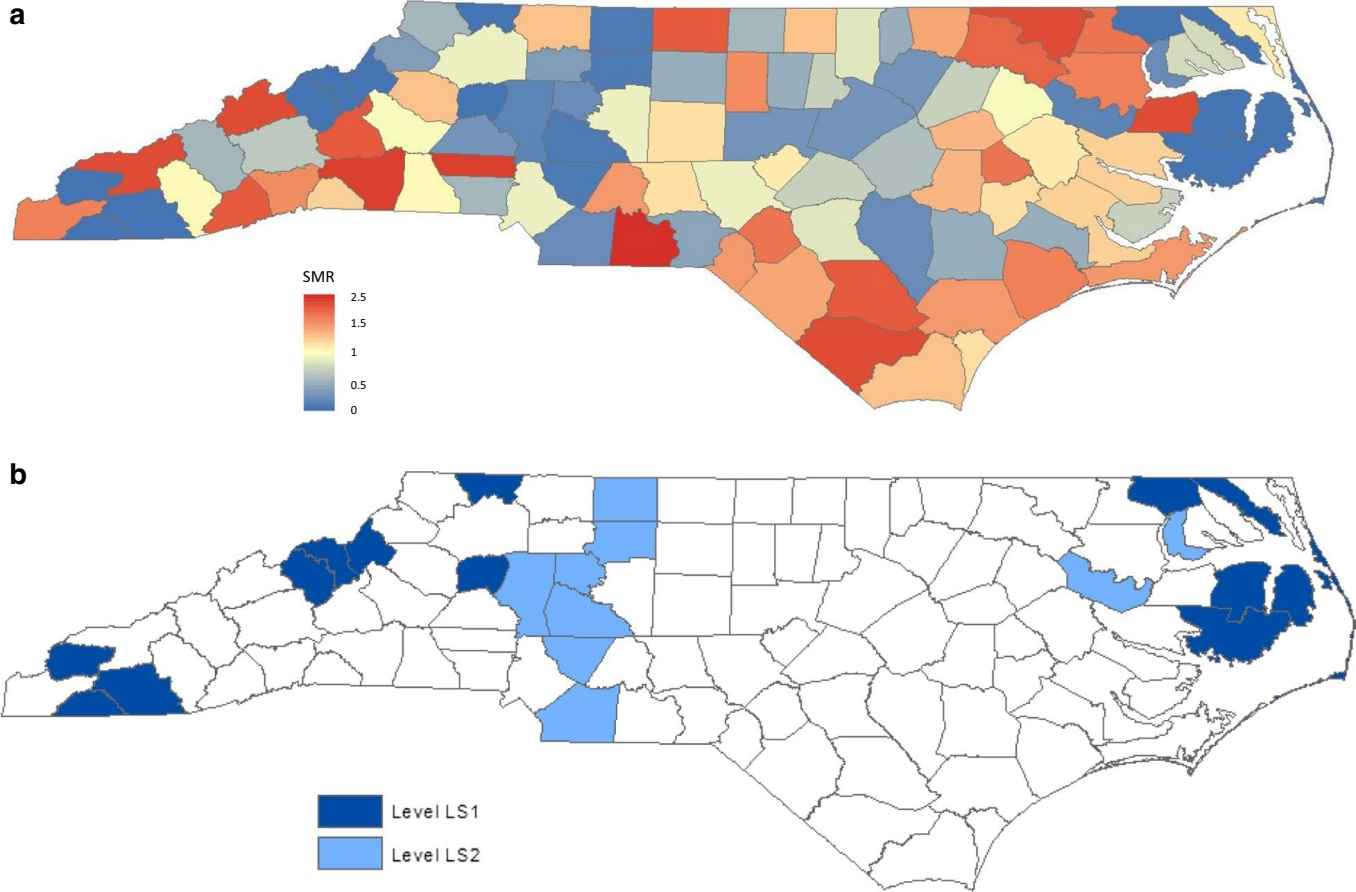
**a** County-specific SMR adjusted-for-race SIDS intensity map in North Carolina. **b** County-Specific SMR adjusted-for-race SIDS intensity-level map in North Carolina

Table 4 presents the rank order and cluster statistics for 25 counties with the highest rates of SMR. The test statistic ***B***_***1***_ in Table 4 was smaller than the ***B***_***1***_ in Table 2 for each on rank order, suggesting that the counties with the higher SMR appear relatively disperse, compared with the counties with the higher raw incidence rates. Figure 2a presents the county-specific SMR-adjusted intensity map.

**Table 4:** Cluster statistics for counties with the higher SMR

Rank order	County	SMR	SIDS	E(SIDS)	B_1_	*P* value^1^	B_2_	*P* value^2^
1	Anson	3.45	15	4.35	–		–	
2	Rutherford	2.47	12	4.86	0	1	0	1
3	Lincoln	2.33	8	3.43	0	1	0	1
4	Madison	2.16	2	0.92	0	1	0	1
5	Northampton	2.01	9	4.47	0	1	0	1
6	Washington	1.97	5	2.54	0	1	0	1
7	Swain	1.95	3	1.54	0	1	0	1
8	Columbus	1.94	15	7.72	0	1	0	1
9	Bladen	1.88	8	4.26	1	$\text{8.61}{\times10}^{-1}$	1	$\text{7.89}{\times10}^{-1}$
10	McDowell	1.874	5	2.67	2	$\text{6.73}{\times10}^{-1}$	2	$\text{5.42}{\times10}^{-1}$
11	Rockingham	1.873	16	8.54	2	$\text{7.83}{\times10}^{-1}$	2	$\text{6.59}{\times10}^{-1}$
12	Transylvania	1.83	3	1.64	2	$\text{8.67}{\times10}^{-1}$	2	$\text{7.60}{\times10}^{-1}$
13	Halifax	1.72	18	10.46	3	$\text{7.67}{\times10}^{-1}$	3	$\text{6.10}{\times10}^{-1}$
14	Hertford	1.66	7	4.22	4	$\text{6.78}{\times10}^{-1}$	4	$\text{4.88}{\times10}^{-1}$
15	Greene	1.65	4	2.43	4	$\text{7.90}{\times10}^{-1}$	4	$\text{6.10}{\times10}^{-1}$
16	Hoke	1.61	7	4.35	4	$\text{8.75}{\times10}^{-1}$	4	$\text{7.22}{\times10}^{-1}$
17	Cherokee	1.53	2	1.31	5	$\text{8.33}{\times10}^{-1}$	4	$\text{8.14}{\times10}^{-1}$
18	Onslow	1.52	29	19.08	5	$\text{9.05}{\times10}^{-1}$	4	$\text{8.84}{\times10}^{-1}$
19	Bertie	1.51	6	3.98	9	$\text{4.70}{\times10}^{-1}$	6	$\text{6.91}{\times10}^{-1}$
20	Alamance	1.48	13	8.81	10	$\text{4.64}{\times10}^{-1}$	6	$\text{7.92}{\times10}^{-1}$
21	Henderson	1.44	5	3.48	12	$\text{3.34}{\times10}^{-1}$	8	$\text{6.06}{\times10}^{-1}$
22	Scotland	1.37	8	5.83	13	$\text{3.45}{\times10}^{-1}$	9	$\text{5.76}{\times10}^{-1}$
23	Pender	1.34	4	2.97	16	$\text{1.72}{\times10}^{-1}$	9	$\text{6.95}{\times10}^{-1}$
24	Carteret	1.328	5	3.766	17	$\text{1.91}{\times10}^{-1}$	9	$\text{7.96}{\times10}^{-1}$
25	Stanly	1.325	5	3.772	18	$\text{2.13}{\times10}^{-1}$	10	$\text{7.82}{\times10}^{-1}$

Previously detected geographical high-intensity clusters in the northeast and south appeared less intensive in terms of rank order and smaller in size, after adjusting for race. It indicates that the high percentage of non-white births alone is not sufficient to explain the excess of SIDS risk thoroughly in the northeastern and southern regions. In addition, the previous cluster in the mid-east disappeared after adjusting for race. On the other side, a small area, which was hidden previously, emerged in the southwest, comprising Rutherford (2nd in rank), McDowell (10th in rank), Transylvania (12th in rank), and Henderson (21st in rank) counties. The raw incidence and SMR of these 4 counties combined are 2.88 per 1000 live births and 1.977, respectively. It indicates that an excess of SIDS risk is observed in this region, after adjusting for race. The *p* values of ***B***_***1***_ for the 25 top ranking counties do not appear cyclic over the rates, and no hierarchy in high SMR-adjusted intensity would be recognized.

**Fig. 3:**
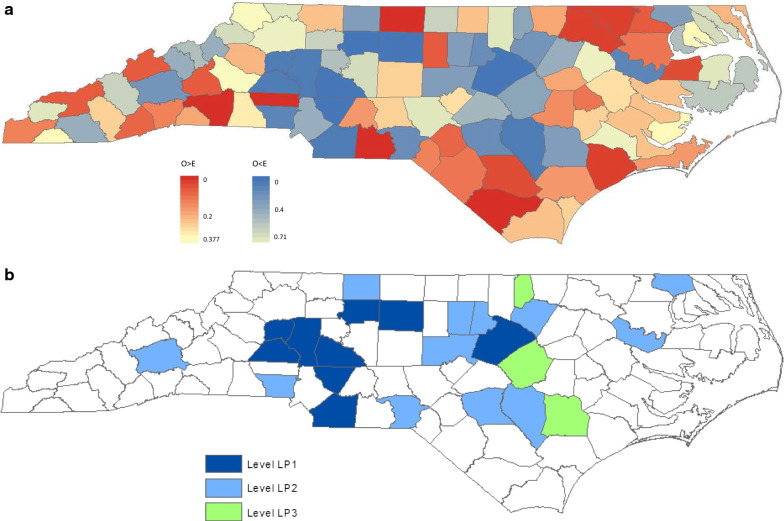
**a** County-specific Poisson adjusted-for-race SIDS intensity map in North Carolina. **b** County-specific Poisson adjusted-for-race SIDS intensity-level map in North Carolina

In the investigation of the geographical disease clustering pattern of low SMR-adjusted intensity, we present the rank order and cluster statistics for 25 counties with the lowest SMR in Table 5. The discrimination between the 13 counties with 0 SIDS was based on the race-adjusted expected numbers of SIDS. The larger the covariate-adjusted expected incidence, the higher in rank order the county will be. The large downward peaks in *p* values relative to surrounding *p* values occurred at including Clay (88th in rank) and Davie (79th in rank) counties, shown in the *p* value^1^ column of Table 5. We, thus, determined the 2 groups of counties to use in constructing hierarchical clusters of mutually neighboring race-adjusted low-risk counties beyond the effect of race: Level-LS1 counties for the 13 top ranking counties with 0 SIDS; Level-LS2 for the 9 counties ranking from 87 to 79, as shown in Fig. 3b. The geographical disease clustering pattern of low SMR-adjusted intensity, shown in Fig. 2a, b, does not appear very different from the geographical clustering pattern of low incidence intensity without adjustment, shown in Fig. 1a, b. One major reason was that the 13 counties with 0 SIDS were among the 13 top ranking counties in both settings. The use of SMR does not seem to effectively discriminate between the 13 counties with 0 SIDS.

**Table 5:** Cluster statistics for counties with the lower SMR

Rank order	County	SMR	SIDS		E(SIDS)	Live birth	B_1_	*P* value^1^	Risk level	B_2_	*P* value^2^
100	Alexander	0	0	1.92		1333	-		LS1	-	
99	Gates	0	0	1.16		420	0	1	LS1	0	1
98	Macon	0	0	0.97		797	0	1	LS1	0	1
97	Yancey	0	0	0.95		770	0	1	LS1	0	1
96	Avery	0	0	0.94		781	0	1	LS1	1	$\text{3.41}{\times10}^{-1}$
95	Mitchell	0	0	0.80		671	2	$\text{1.53}{\times10}^{-1}$	LS1	3	$\text{2.04}{\times10}^{-2}$
94	Hyde	0	0	0.75		338	2	$\text{2.65}{\times10}^{-1}$	LS1	3	$\text{4.57}{\times10}^{-2}$
93	Dare	0	0	0.73		521	3	$\text{1.47}{\times10}^{-1}$	LS1	3	$\text{9.06}{\times10}^{-2}$
92	Camden	0	0	0.64		286	4	$\text{9.29}{\times10}^{-2}$	LS1	3	$\text{1.59}{\times10}^{-1}$
91	Alleghany	0	0	0.61		487	4	$\text{1.70}{\times10}^{-1}$	LS1	3	$\text{2.53}{\times10}^{-1}$
90	Graham	0	0	0.599		415	5	$\text{1.26}{\times10}^{-1}$	LS1	4	$\text{1.62}{\times10}^{-1}$
89	Tyrrell	0	0	0.598		248	6	$\text{9.92}{\times10}^{-2}$	LS1	5	$\text{1.11}{\times10}^{-1}$
88	Clay	0	0	0.34		284	7	$\text{8.36}{\times10}^{-2}$	LS1	7	$\text{3.31}{\times10}^{-2}$
87	Rowan	0.36	3	8.24		4606	7	$\text{1.52}{\times10}^{-1}$	LS2	7	$\text{6.33}{\times10}^{-2}$
86	Forsyth	0.41	10	24.34		11,858	7	$\text{2.50}{\times10}^{-1}$	LS2	7	$\text{1.14}{\times10}^{-1}$
85	Cabarrus	0.42	3	7.12		4099	8	$\text{2.30}{\times10}^{-1}$	LS2	8	$\text{9.45}{\times10}^{-2}$
84	Stokes	0.43	1	2.34		1612	9	$\text{2.17}{\times10}^{-1}$	LS2	9	$\text{8.30}{\times10}^{-2}$
83	Martin	0.48	2	4.15		1549	9	$\text{3.34}{\times10}^{-1}$	LS2	9	$\text{1.42}{\times10}^{-1}$
82	Iredell	0.51	4	7.91		4139	12	$\text{1.28}{\times10}^{-1}$	LS2	11	$\text{6.99}{\times10}^{-2}$
81	Chowan	0.539	1	1.85		751	13	$\text{1.34}{\times10}^{-1}$	LS2	14	$\text{1.70}{\times10}^{-2}$
80	Union	0.543	4	7.36		3915	14	$\text{1.43}{\times10}^{-1}$	LS2	15	$\text{1.78}{\times10}^{-2}$
79	Davie	0.548	1	1.82		1207	17	$\text{5.72}{\times10}^{-2}$	LS2	18	$\text{4.53}{\times10}^{-3}$
78	Sampson	0.552	4	7.24		3025	17	$\text{1.10}{\times10}^{-1}$		18	$\text{1.03}{\times10}^{-2}$
77	Wake	0.557	16	28.72		14,484	17	$\text{1.91}{\times10}^{-1}$		18	$\text{2.24}{\times10}^{-2}$
76	Franklin	0.558	2	3.58		1399	18	$\text{2.13}{\times10}^{-1}$		19	$\text{2.47}{\times10}^{-2}$

### Poisson-model with adjustment for race

We list the counties in rank order based on ***D***_***i***_ and present the cluster statistics for 25 top ranking counties with the smallest ***D***_***i***_ and *N*_*i*_* ≥ μ(A*_*i*_*)* in Table 6. The 10 top ranking counties in Table 6 had significantly higher incidence than the expected race-adjusted incidence each at a nominal significance level of 0.05 under the assumption of Poisson distribution, suggesting that the departure from expected race-adjusted incidence for each of these 10 counties is too large to be attributed to chance alone. All but 2 top ranking counties in Table 6 also appear on the list of the 25 top ranking counties with the highest SMR in Table 4, except Robeson (18th in rank) and Wayne (24th in rank) in Table 6 vs. Pender (23rd in rank) and Stanly (25th in rank) in Table 4. So the geographical race-adjusted high-intensity clustering patterns, characterized by the Poisson model and SMR, are similar, shown in Figs. 2a and 3a. The smaller the value of ***D***_***i***_ with *N*_*i*_* ≥ μ(A*_*i*_*)*, the darker in red the county is, shown in Fig. 3a. The conclusion based on the Poisson model was similar to the one based on the higher SMR rates. The *p* values of ***B***_***1***_ for these 25 top ranking counties do not appear cyclic. No hierarchy in high race-adjusted intensity would be recognized beyond the effect of race by the Poisson model.

**Table 6:** Cluster statistics for counties with the smaller poisson probability for *N*_*i*_* ≥ μ(A*_*i*_*)*

Rank order	County	SMR	SIDS	E(SIDS)	Poisson	B_1_	*P* value^1^	B_2_	*P* value^2^
1	Anson	3.45	15	4.35	$\text{1.36}{\times10}^{-5}$	–		–	
2	Rutherford	2.47	12	4.86	$\text{1.57}{\times10}^{-3}$	0	1	0	1
3	Columbus	1.94	15	7.72	$\text{6.00}{\times10}^{-3}$	0	1	0	1
4	Rockingham	1.87	16	8.54	$\text{6.92}{\times10}^{-3}$	0	1	0	1
5	Lincoln	2.33	8	3.43	$\text{8.72}{\times10}^{-3}$	0	1	0	1
6	Halifax	1.72	18	10.46	$\text{1.11}{\times10}^{-2}$	0	1	0	1
7	Onslow	1.52	29	19.08	$\text{1.24}{\times10}^{-2}$	0	1	0	1
8	Northampton	2.01	9	4.47	$\text{1.64}{\times10}^{-2}$	1	$\text{7.81}{\times10}^{-1}$	1	$\text{6.98}{\times10}^{-1}$
9	Bladen	1.88	8	4.26	$\text{3.00}{\times10}^{-2}$	2	$\text{5.40}{\times10}^{-1}$	2	$\text{4.17}{\times10}^{-1}$
10	Washington	1.97	5	2.54	$\text{4.46}{\times10}^{-2}$	2	$\text{6.73}{\times10}^{-1}$	2	$\text{5.42}{\times10}^{-1}$
11	McDowell	1.87	5	2.67	$\text{5.43}{\times10}^{-2}$	3	$\text{5.16}{\times10}^{-1}$	3	$\text{3.65}{\times10}^{-1}$
12	Alamance	1.48	13	8.81	$\text{6.45}{\times10}^{-2}$	4	$\text{4.05}{\times10}^{-1}$	3	$\text{4.88}{\times10}^{-1}$
13	Hertford	1.66	7	4.22	$\text{6.50}{\times10}^{-2}$	5	$\text{3.32}{\times10}^{-1}$	4	$\text{3.65}{\times10}^{-1}$
14	Madison	2.16	2	0.92	$\text{6.70}{\times10}^{-2}$	5	$\text{4.67}{\times10}^{-1}$	4	$\text{4.88}{\times10}^{-1}$
15	Swain	1.95	3	1.54	$\text{7.03}{\times10}^{-2}$	5	$\text{6.05}{\times10}^{-1}$	4	$\text{6.10}{\times10}^{-1}$
16	Hoke	1.61	7	4.35	$\text{7.49}{\times10}^{-2}$	5	$\text{7.32}{\times10}^{-1}$	4	$\text{7.22}{\times10}^{-1}$
17	Transylvania	1.83	3	1.64	$\text{8.41}{\times10}^{-2}$	5	$\text{8.33}{\times10}^{-1}$	4	$\text{8.14}{\times10}^{-1}$
18	Robeson	1.25	31	24.78	$\text{9.23}{\times10}^{-2}$	8	$\text{4.86}{\times10}^{-1}$	7	$\text{3.96}{\times10}^{-1}$
19	Greene	1.65	4	2.43	$\text{9.94}{\times10}^{-2}$	8	$\text{6.25}{\times10}^{-1}$	7	$\text{5.21}{\times10}^{-1}$
20	Bertie	1.51	6	3.98	$\text{1.08}{\times10}^{-1}$	12	$\text{2.17}{\times10}^{-1}$	9	$\text{3.29}{\times10}^{-1}$
21	Scotland	1.37	8	5.83	$\text{1.36}{\times10}^{-1}$	14	$\text{1.43}{\times10}^{-1}$	11	$\text{1.97}{\times10}^{-1}$
22	Henderson	1.44	5	3.48	$\text{1.40}{\times10}^{-1}$	16	$\text{9.69}{\times10}^{-2}$	13	$\text{1.16}{\times10}^{-1}$
23	Cherokee	1.53	2	1.31	$\text{1.45}{\times10}^{-1}$	17	$\text{1.10}{\times10}^{-1}$	13	$\text{1.89}{\times10}^{-1}$
24	Wayne	1.23	18	14.67	$\text{1.58}{\times10}^{-1}$	18	$\text{1.26}{\times10}^{-1}$	14	$\text{1.90}{\times10}^{-1}$
25	Carteret	1.33	5	3.77	$\text{1.79}{\times10}^{-1}$	19	$\text{1.44}{\times10}^{-1}$	14	$\text{2.89}{\times10}^{-1}$

While the geographical race-adjusted low-intensity clustering pattern based on the Poisson model appears very different from the one based on the low SMR rates. The smaller the value of ***D***_***i***_ with *N*_*i*_* ≤ μ(A*_*i*_*)*, the darker in blue the county is, shown in Fig. 3a. There were only 3 counties (Alexander, Gates, and Macon) with 0 SIDS among the 25 top ranking counties with the smallest ***D***_***i***_ and *N*_*i*_* ≤ μ(A*_*i*_*)* in Table 7. Although only the 4 top ranking counties had significantly lower incidence than the race-adjusted expected incidence each at a nominal significance level of 0.05, it remained meaningful and useful to search for and determine regions of several (or many) mutually adjacent counties with the low race-adjusted rates geographically, beyond the effect of race. Regions of similar low incidence may reveal information on the presence of protective factors or the absence of risk factors associated with SIDS.

**Table 7:** Cluster statistics for counties with the smaller poisson probability for *N*_*i*_* ≤ μ(A*_*i*_*)*

Rank order	County	SMR	SIDS	E(SIDS)	Poisson	B_1_	*P* value^1^	Risk level	B_2_	*P* value^2^
100	Forsyth	0.41	10	24.34	$\text{8.80}{\times10}^{-4}$	–		LP1	–	
99	Wake	0.56	16	28.72	$\text{7.23}{\times10}^{-3}$	0	1	LP1	0	1
98	Guilford	0.69	23	33.57	$\text{3.53}{\times10}^{-2}$	1	$\text{1.43}{\times10}^{-1}$	LP1	1	$\text{1.16}{\times10}^{-1}$
97	Rowan	0.36	3	8.24	$\text{3.59}{\times10}^{-2}$	1	$\text{2.68}{\times10}^{-1}$	LP1	1	$\text{2.20}{\times10}^{-1}$
96	Cabarrus	0.42	3	7.12	$\text{7.59}{\times10}^{-2}$	2	$\text{7.27}{\times10}^{-2}$	LP1	2	$\text{4.87}{\times10}^{-2}$
95	Iredell	0.51	4	7.91	$\text{1.05}{\times10}^{-1}$	4	$\text{6.70}{\times10}^{-3}$	LP1	3	$\text{2.04}{\times10}^{-2}$
94	Catawba	0.56	5	8.92	$\text{1.21}{\times10}^{-1}$	5	$\text{4.36}{\times10}^{-3}$	LP1	4	$\text{9.59}{\times10}^{-3}$
93	Union	0.54	4	7.36	$\text{1.43}{\times10}^{-1}$	6	$\text{3.21}{\times10}^{-3}$	LP1	5	$\text{5.45}{\times10}^{-3}$
92	Alexander	0.00	0	1.92	$\text{1.46}{\times10}^{-1}$	8	$\text{6.85}{\times10}^{-4}$	LP1	7	$\text{8.79}{\times10}^{-4}$
91	Sampson	0.55	4	7.24	$\text{1.52}{\times10}^{-1}$	8	$\text{2.25}{\times10}^{-3}$	LP2	7	$\text{2.52}{\times10}^{-3}$
90	Gaston	0.75	11	14.71	$\text{2.05}{\times10}^{-1}$	8	$\text{6.44}{\times10}^{-3}$	LP2	8	$\text{1.98}{\times10}^{-3}$
89	Martin	0.48	2	4.15	$\text{2.17}{\times10}^{-1}$	8	$\text{1.61}{\times10}^{-2}$	LP2	8	$\text{5.24}{\times10}^{-3}$
88	Cumberland	0.89	38	42.62	$\text{2.69}{\times10}^{-1}$	9	$\text{1.46}{\times10}^{-2}$	LP2	9	$\text{4.46}{\times10}^{-3}$
87	Durham	0.83	16	19.22	$\text{2.75}{\times10}^{-1}$	10	$\text{1.40}{\times10}^{-2}$	LP2	10	$\text{3.80}{\times10}^{-3}$
86	Richmond	0.67	4	6.00	$\text{2.85}{\times10}^{-1}$	10	$\text{3.25}{\times10}^{-2}$	LP2	10	$\text{8.98}{\times10}^{-3}$
85	Buncombe	0.79	9	11.38	$\text{3.01}{\times10}^{-1}$	10	$\text{6.75}{\times10}^{-2}$	LP2	10	$\text{1.94}{\times10}^{-2}$
84	Franklin	0.56	2	3.58	$\text{3.05}{\times10}^{-1}$	11	$\text{6.68}{\times10}^{-2}$	LP2	11	$\text{1.80}{\times10}^{-2}$
83	Gates	0.00	0	1.16	$\text{3.13}{\times10}^{-1}$	11	$\text{1.25}{\times10}^{-1}$	LP2	11	$\text{3.68}{\times10}^{-2}$
82	Orange	0.69	4	5.79	$\text{3.14}{\times10}^{-1}$	12	$\text{1.28}{\times10}^{-1}$	LP2	12	$\text{3.53}{\times10}^{-2}$
81	Chatham	0.57	2	3.50	$\text{3.21}{\times10}^{-1}$	15	$\text{4.36}{\times10}^{-2}$	LP2	15	$\text{8.03}{\times10}^{-3}$
80	Stokes	0.43	1	2.34	$\text{3.22}{\times10}^{-1}$	17	$\text{2.70}{\times10}^{-2}$	LP2	16	$\text{8.58}{\times10}^{-3}$
79	Duplin	0.70	4	5.72	$\text{3.24}{\times10}^{-1}$	18	$\text{3.22}{\times10}^{-2}$	LP3	17	$\text{9.38}{\times10}^{-3}$
78	Vance	0.71	4	5.67	$\text{3.32}{\times10}^{-1}$	19	$\text{3.89}{\times10}^{-2}$	LP3	18	$\text{1.03}{\times10}^{-2}$
77	Johnston	0.77	6	7.80	$\text{3.38}{\times10}^{-1}$	22	$\text{1.51}{\times10}^{-2}$	LP3	19	$\text{1.19}{\times10}^{-2}$
76	Macon	0.00	0	0.97	$\text{3.78}{\times10}^{-1}$	22	$\text{3.45}{\times10}^{-2}$		19	$\text{2.47}{\times10}^{-2}$

The large downward peaks in *p* values of ***B***_***1***_ relative to surrounding *p* values occurred at including Alexander (92nd in rank), Stokes (80th in rank), and Johnson (77th in rank) counties, shown in the *p* value^1^ column of Table 7. We determined the 3 groups of counties to use in constructing hierarchical clusters of mutually neighboring race-adjusted low-risk counties with 3 different levels of intensity, after adjusting for the effect of race. Level-LP1 counties are the 9 top ranking counties (92nd–100th in rank); Level-LP2, 12 counties ranking from 80 to 91; Level-LP3, 3 counties ranking from 77 to 79. The raw incidence of the 9 Level-LP1, 12 Level-LP2, and 3 Level-LP3 counties are 0.85, 1.24, and1.65 per 1000 live births, respectively. The 3 hierarchical race-adjusted low-intensity clusters appear in the north (3 counties: 2 Level-LP1 and 1 Level-LP2) with raw incidence of 0.96, the mid-west (6 Level-LP1 counties) with raw incidence of 0.71, and the mid-east (10 counties: 1 Level-LP1, 6 Level-LP2, and 3 Level-LP3) with raw incidence of 1.40 per 1000 live births. Figure 3b presents this county-specific intensity-level map, by the Poisson model.

Our analysis showed that the difference in racial distribution of live births across North Carolina explained, to a large extent, the 3 previously identified hierarchical high-intensity clusters in the northeast, south, and mid-east, shown in Fig. 1a, b. None *p* value of ***B***_***1***_ for all *k* was statistically significant at a nominal significance level of 0.05 in the testing for the presence of clustering of the counties with the higher race-adjusted incidence intensity, characterized by both the models. It was because these 25 top ranking counties with the highest adjusted intensity rates in the settings of either models were allocated sequentially and alternatively across the 3 relatively high adjusted-intensity regions in the northeast, south, and southwest, shown in Figs. 2a and 3a. It indicates that the counties with the higher race-adjusted rates did not show high degree of clustering geographically. No hierarchy in high race-adjusted intensity would be recognized.

Intriguingly, a small region of 4 mutually adjacent counties with the higher race-adjusted rates, which was hidden previously, emerged in the southwest. The combined raw incidence and SMR of this region are 2.88 per 1000 live births and 1.977, respectively. The combined SMR of 1.977 in this small region was higher than the 95 of the 100 counties, shown in Table 4. Unobserved spatially related risk factors may cause the elevated risk in this region.

In contrast, the Poisson model appeared more appropriate than the model based on SMR in the study of geographical race-adjusted low-intensity clustering patterns, particularly at facilitating discrimination between the 13 counties with 0 SIDS but different numbers of live births. In the comparison of Fig. 1a, b with Fig. 3a, b, uneven distribution of the race-specific live births substantially changed the geographical race-adjusted low-intensity clustering patterns, too. The previously detected geographical low-intensity clusters in the northwest and the eastern coast disappeared, indicating that high percentage of white births alone was sufficient to explain the excess of SIDS risk in these 2 regions. The previously detected geographical low-intensity cluster in the mid-west remained, but divided into 2 smaller distinct geographical clusters. A large geographical cluster of 10 mutually adjacent counties with the low race-adjusted rates, which was hidden previously, emerged in the mid-east. It suggests that unidentified spatially related protective factors could explain the unusually low-risk clusters in the mid-west and the mid-east. A summary of hierarchical cluster analysis based on different models is presented in Table 8.

**Table 8:** Summary of hierarchical cluster analysis by different models

Risk	Without adjustment	Adjustment for race	
		SMR model	Poisson model
Higher rates	Northeast (6 counties: 5 Level-H1, 1 Level-H2) with incidence of 4.98 South (6 counties: 1 Level-H1, 5 Level-H2) with incidence of 4.06 Mid-East (6 counties: 1 Level-H1, 5 Level-H3) with incidence of 3.09	None	None
Lower rates	East (3 counties: 3 Level-L1) with incidence of 0.0 Northwest (6 counties: 4 Level-L1, 2 Level-L2) with incidence of 0.28 Mid-West (9 counties: 1 Level-L1, 8 Level-L2) with incidence of 0.70	East (3 counties: 3 Level-LS1) with incidence of 0.0 Northwest (3 counties: 3 Level-LS1) with incidence of 0.0 Mid-West (8 counties: 1 Level-LS1, 7 Level-LS2) with incidence of 0.79	Mid-West (6 counties: 6 Level-LP1) with incidence of 0.71 North (3 counties: 2 Level-LP1, 1 Level-LP2) with incidence of 0.96 Mid-East (10 counties: 1 Level-LP1, 6 Level-LP2, 3 Level-LP2) with incidence of 1.40

The incidence rate in this table indicates the value of raw incidence per 1000 live births

### Analysis with different neighborhood systems

The definition of neighborhood systems may govern the analysis outcomes. Because of the irregularity in shape, contour, size, and location among distinct areas under study, special spatial configurations of certain sampled areas may be intuitively clustering, but not all of which are actually adjacent geographically. Due to the inherent irregular nature of most spatial data, we consider two distinct neighborhood systems for the North Carolina counties in illustrating the spatial statistical models that we propose in this report. In addition to the one based on geographical adjacency that we have used previously, we extended the proposed methods by using a distance-based neighborhood system, in which the neighbor of counties is defined with being within 30 miles between the seats of the 2 counties in this application. The neighborhood information based on this criterion is available in the existing literature [4].

We propose the use of a distance-based definition of neighbors as follows: within 30 miles between the seats of the 2 counties, denoted by ***ω***^***2***^, in this application. We carried out the analysis with the test statistic ***B***_***2***_** = (1/2) Σ ω**^***2***^_***ij***_*** x***_***i***_*** x***_***j***_, where ***x***_***i***_** = 1** if county ***i*** is a high-risk (or low-risk) county for some disease and ***0*** otherwise, and where ***ω***^***2***^_***ij***_** = 1 **if counties ***i ***and* j* whose seats are closer less than 30 miles and ***0*** otherwise, ***ω***^***2***^_***ij***_** = ω**^***2***^_***ji***_,* ω*^***2***^_***ii***_** = 0**. The sum ranges over all pairs of counties. Again, we used simulation-based permutations using 1 million replicates based on ***ω***^***2***^ and obtained the null distribution of ***B***_***2***_. Each of the 24 distributions of ***B***_***2***_ for *k* = 2, 3, 4… 25 is given in Additional file 1. Table S1. The values of the test statistic ***B***_***2***_ and the associated *p* value, denoted by *p* value^2^, are presented on the right panel of Tables 2, 3, 4, 5, 6 and7.

The geographical incidence intensity clustering patterns characterized by ***B***_***2***_ and *p* value^2^ were generally in close agreement with those by ***B***_***1***_ and *p* value^1^ with and without the adjustment for race. It is not surprising in this particular application as the neighborhood system, defined using the 30-mile criterion, corresponds nearly precisely to the one, defined by those counties mutually sharing common geographical boundaries, in North Carolina county system [4, 5].

### Comparison with analysis using spatial scan statistic

Both our proposed statistical methods and the spatial scan statistic allow for confounding variables and are used to identify disease clustering or detect disease clusters in a spatial point process in general. They are sensitive to different respects of spatially characteristic incidence clustering patterns and structured to provide different spatial clustering information. The spatial scan statistic determines the most likely disease clusters and secondary disease clusters based on the maximum likelihood ratio, whose statistical significance is evaluated, using Monte Carlo hypothesis testing. The spatial scan statistic tends to detect relatively broad spatial clusters, and the detected most likely disease clusters may not be the regions with the highest rates. In the analysis of SIDS in North Carolina counties, the spatial scan statistic detected the most likely cluster in the south with incidence of 3.8 and the secondary cluster in the northeast with incidence of 4.1 per 1000 live births, with the state-wide incidence of 2.0, shown in Table 1 [8].

In contrast, our proposed methods are designed to prioritize the counties with the highest or lowest, adjusted or unadjusted, intensity rates in the testing for the presence of spatial clustering. We let the threshold rate of incidence vary downwards continuously, in which case it includes exactly next one area in the rank order at one time, up to a certain upper limit. As shown in Table 2 and Figs. 1a, b, the highest intensive clustering region was recognized in the northeast with incidence of 4.98, and secondary intensive clustering region, in the south with incidence of 4.06 per 1, 00 live births.

After adjusting for race, the spatial scan statistic detected an emerging broad secondary cluster in the west, which was previously hidden and comprised 18 counties, with the SMR of 1.357, presented in Table 2 [8]. In contrast, our method, either based on the Poisson model or SMR, recognized a small high-intensity region, which was previously hidden and emerged in the southwest, comprising only 4 counties: Rutherford, McDowell, Transylvania, and Henderson, shown in Tables 4 and 6 and Figs. 2a and 3a. This small regions in the southwest was a much smaller subset of the broad region identified by the spatial scan statistic and had a higher SMR of 1.977 per 1000 live births.

## Discussion

In this paper, we have presented a general framework for differentiating intensity of geographical disease clusters of peak incidence and low incidence in a hierarchical manner with the adjustment for covariates as well as testing for the presence of disease clustering. The first method is structured for recognizing and constructing hierarchical (in intensity) disease clusters of low incidence. The second method generalizes to take into account covariates that are known or hypothesized risk factors of the disease in constructing hierarchical (in adjusted intensity) clusters of high-risk areas and low-risk areas close within geographic proximity, respectively. We formulated the adjustment for covariates by calculating the expected number of cases in each county through indirect standardization. We proposed two probability models, a Poisson-distribution-model and SMR, to facilitate discrimination between the 100 North Carolina counties based on the deviation between the observed incidence and covariate-adjusted expected incidence in each county, through which the hierarchy in adjusted intensity is recognized, beyond the effect of covariates.

The application to the data on North Carolina SIDS, using the proposed methods, shows that the two probability models performed similarly in the geographical race-adjusted intensity clustering analysis of counties with the highest rates. While the analysis was very different in the investigation of the mutually adjacent counties with the lowest adjusted rates. The Poisson model that can account for the unequal variances of the county rates performed better particularly at facilitating discrimination between the 13 counties with zero SIDS but different numbers of live births than the model based on SMR. The hierarchical race-adjusted low-intensity clusters, characterized by the Poisson model, should be more reliable, shown in Tables 6 and 7 and Fig. 3a, b.

With the information on hierarchy in adjusted intensity levels, provided by the application of our proposed methods, epidemiologists can best prioritize the regions with the highest rates within which to conduct thorough etiologic investigations and search for hidden spatially related risk factors. Similar research designs are commonly applied in studies of human genetics, in which a group of affected sibships with extreme traits are used for detecting commonly shared genetic defects of a disease of interest in gene mappings [15]. Meanwhile, public health officials can better prioritize the high-risk regions precisely and promptly move resources to areas with genuine highest abnormalities.

The identification of geographical and temporal disease clusters serves as a preliminary step that expedites subsequent etiological investigation and analysis of epidemicity. Most reports of perceived clusters do not lead to the identification of a common casual exposure for the events of interest [16]. The reasons for this are many. As Rothman and many others pointed out that vast resources spent on the investigation of possible alarms of disease clustering are often in vain. We should not be aiming to detect clustering, but to understand why clusters occur [17]. The four stages in the guidelines for the investigation of disease clusters issued by the US Centers for Disease Control in 1990 are (1) initial contact with and response to the individual who reported the cluster; (2) a preliminary assessment, including evaluations of whether an excess has occurred; (3) a formal feasibility study; and (4) a full etiologic investigation [18]. The ordinary statistical methods for detecting temporal and spatial clustering in disease incidence frequency alone are useful in the second stage. While, the utilities of our proposed statistical methods contribute to the third and fourth stages.

In addition to the focus on peak incidence, we extended the proposed methods to investigate geographical disease clusters of low incidence. We exemplified the utilities of recognizing and constructing the geographical hierarchical (in intensity) disease clusters of low incidence and peak incidence without and with the adjustment for covariates. The studies of incidence paucity and incidence clustering characterize opposite aspects of an observed geographical incidence pattern by using different parts of information from the data. In this report, we show that statistical methods that focus on geographical incidence paucity can be as meaningful and useful in spatial epidemiology and spatial statistics as the methods that focus on peak incidence in space. We articulate the difference in sensitivity, power, and applicability between the studies of incidence paucity and incidence clustering, using a temporal series of data, in our previous articles [1, 2].

## Supplementary information

**Additional file 1: Table S1.** Frequency distributions of the number of neighbors (seats of counties within 30 miles) simulated on the basis of 1 million random selections in North Carolina counties.

## Data Availability

Information on data on the spatial occurrence of SIDS in North Carolina counties from July 01, 1974 to June 30, 1978, is available on the paper: Cressie N, Chan NH (1989). Spatial Modeling of Regional Variables. *Journal of the American Statistical Association*, 84(406):393–401.
