# Supplementary material for: Differentiating anomalous disease intensity with confounding variables in space
Source: Int J Health Geogr. 2020 Sep 14;19:37. doi: 10.1186/s12942-020-00231-3 (PMC7489047; doi:10.1186/s12942-020-00231-3)
Supplement: Supplementary file 1 — Additional file 1: Table S1. Frequency distributions of the number of neighbors (seats of counties within 30 miles) simulated on the basis of 1 million random selections in North Carolina counties. [file 12942_2020_231_MOESM1_ESM.docx]

| Supplementary Table 1: Frequency Distributions of the Number of Neighbors (Seats of Counties within 30 Miles) Simulated on the Basis of 1 Million Random Selections in North Carolina Counties | | | | | | | | | | | | | | | | | | |
| --- | --- | --- | --- | --- | --- | --- | --- | --- | --- | --- | --- | --- | --- | --- | --- | --- | --- | --- |
| Test Statistic B | | | | | | | | | | | | | | | | | | |
|  | 0 | 1 | 2 | 3 | 4 | 5 | 6 | 7 | 8 | 9 | 10 | 11 | 12 | 13 | 14 | 15 | 16 | 17 |
| Number of risk districts | | | | | | | | | | | | | | | | | | |
| 2 | 960105 | 39895 |  |  |  |  |  |  |  |  |  |  |  |  |  |  |  |  |
| 3 | 884107 | 112703 | 2642 | 548 |  |  |  |  |  |  |  |  |  |  |  |  |  |  |
| 4 | 780103 | 203081 | 14280 | 2409 | 101 | 25 | 1 |  |  |  |  |  |  |  |  |  |  |  |
| 5 | 659129 | 292220 | 41031 | 6741 | 712 | 141 | 24 | 1 | 1 |  |  |  |  |  |  |  |  |  |
| 6 | 532530 | 361863 | 85255 | 17101 | 2650 | 497 | 92 | 9 | 3 |  |  |  |  |  |  |  |  |  |
| 7 | 410730 | 400222 | 143398 | 36057 | 7608 | 1547 | 349 | 69 | 17 | 2 | 1 |  |  |  |  |  |  |  |
| 8 | 302348 | 401613 | 205416 | 67199 | 17979 | 4169 | 1011 | 197 | 55 | 13 |  |  |  |  |  |  |  |  |
| 9 | 211132 | 371649 | 258406 | 108933 | 36006 | 10311 | 2684 | 643 | 182 | 41 | 11 | 2 |  |  |  |  |  |  |
| 10 | 140779 | 317062 | 289271 | 158148 | 64121 | 21522 | 6578 | 1825 | 509 | 136 | 35 | 9 | 3 | 2 |  |  |  |  |
| 11 | 89509 | 251433 | 293765 | 203369 | 100658 | 40550 | 14189 | 4546 | 1407 | 408 | 124 | 29 | 8 | 1 | 3 |  | 1 |  |
| 12 | 53313 | 186529 | 271817 | 234493 | 142617 | 68127 | 27679 | 10190 | 3541 | 1167 | 366 | 105 | 34 | 14 | 6 | 2 |  |  |
| 13 | 30514 | 128397 | 231093 | 244860 | 180244 | 102993 | 48844 | 20677 | 7917 | 2927 | 1010 | 354 | 114 | 32 | 15 | 7 | 1 | 1 |
| 14 | 16156 | 82354 | 181412 | 232143 | 207138 | 139724 | 77725 | 37110 | 16031 | 6405 | 2399 | 905 | 334 | 110 | 35 | 11 | 5 | 1 |
| 15 | 8125 | 49511 | 130565 | 201784 | 213769 | 171535 | 110908 | 61258 | 30150 | 13413 | 5523 | 2176 | 812 | 308 | 113 | 24 | 14 | 8 |
| 16 | 3885 | 27896 | 86852 | 159727 | 201404 | 190842 | 143513 | 91366 | 49920 | 25180 | 11292 | 4877 | 2008 | 784 | 295 | 95 | 43 | 12 |
| 17 | 1811 | 14657 | 53012 | 116121 | 172865 | 191527 | 167884 | 122391 | 76770 | 42983 | 21947 | 10129 | 4706 | 1944 | 774 | 294 | 117 | 43 |
| 18 | 735 | 7011 | 30273 | 77483 | 135716 | 174467 | 178368 | 148554 | 105833 | 66928 | 37818 | 19626 | 9568 | 4374 | 1915 | 815 | 325 | 118 |
| 19 | 305 | 3158 | 16017 | 47541 | 96310 | 145533 | 170187 | 163469 | 133438 | 94206 | 59939 | 34613 | 18280 | 9171 | 4403 | 1994 | 831 | 369 |
| 20 | 110 | 1410 | 7650 | 26905 | 62800 | 109361 | 148273 | 163715 | 150957 | 120607 | 85922 | 55168 | 32504 | 17575 | 9011 | 4343 | 2080 | 952 |
| 21 | 34 | 531 | 3462 | 13550 | 37068 | 75128 | 116773 | 147746 | 155463 | 141315 | 111916 | 79535 | 51654 | 30819 | 17239 | 9183 | 4554 | 2207 |
| 22 | 8 | 178 | 1473 | 6405 | 20339 | 46704 | 83616 | 120580 | 144933 | 147648 | 132045 | 104738 | 75178 | 49738 | 30386 | 17279 | 9368 | 4855 |
| 23 | 3 | 61 | 517 | 2844 | 9855 | 26662 | 54294 | 89407 | 121492 | 140649 | 140536 | 124619 | 99757 | 72481 | 48734 | 30068 | 17774 | 9962 |
| 24 | 1 | 18 | 169 | 1095 | 4467 | 13734 | 32011 | 60079 | 92517 | 119767 | 135669 | 134471 | 119339 | 96434 | 70785 | 48105 | 30298 | 18617 |
| 25 |  | 7 | 57 | 378 | 1856 | 6355 | 17072 | 35767 | 63135 | 92986 | 118282 | 131717 | 129054 | 114756 | 93692 | 69704 | 48918 | 31731 |

| Test Statistic B | | | | | | | | | | | | | | | | | | | |
| --- | --- | --- | --- | --- | --- | --- | --- | --- | --- | --- | --- | --- | --- | --- | --- | --- | --- | --- | --- |
|  | 18 | 19 | 20 | 21 | 22 | 23 | 24 | 25 | 26 | 27 | 28 | 29 | 30 | 31 | 32 | 33 | mean | variance |  |
| Number of risk districts | | | | | | | | | | | | | | | | | | | |
| 2 |  |  |  |  |  |  |  |  |  |  |  |  |  |  |  |  | 0.039895 | 0.038303 |  |
| 3 |  |  |  |  |  |  |  |  |  |  |  |  |  |  |  |  | 0.119631 | 0.113892 |  |
| 4 |  |  |  |  |  |  |  |  |  |  |  |  |  |  |  |  | 0.239403 | 0.226845 |  |
| 5 |  |  |  |  |  |  |  |  |  |  |  |  |  |  |  |  | 0.398217 | 0.374331 |  |
| 6 |  |  |  |  |  |  |  |  |  |  |  |  |  |  |  |  | 0.5974 | 0.558676 |  |
| 7 |  |  |  |  |  |  |  |  |  |  |  |  |  |  |  |  | 0.836097 | 0.776968 |  |
| 8 |  |  |  |  |  |  |  |  |  |  |  |  |  |  |  |  | 1.114805 | 1.02779 |  |
| 9 |  |  |  |  |  |  |  |  |  |  |  |  |  |  |  |  | 1.433401 | 1.309346 |  |
| 10 |  |  |  |  |  |  |  |  |  |  |  |  |  |  |  |  | 1.792192 | 1.624697 |  |
| 11 |  |  |  |  |  |  |  |  |  |  |  |  |  |  |  |  | 2.188062 | 1.968207 |  |
| 12 |  |  |  |  |  |  |  |  |  |  |  |  |  |  |  |  | 2.626499 | 2.345884 |  |
| 13 |  |  |  |  |  |  |  |  |  |  |  |  |  |  |  |  | 3.104712 | 2.762054 |  |
| 14 | 2 |  |  |  |  |  |  |  |  |  |  |  |  |  |  |  | 3.620963 | 3.182149 |  |
| 15 | 3 |  | 1 |  |  |  |  |  |  |  |  |  |  |  |  |  | 4.180205 | 3.652171 |  |
| 16 | 6 | 2 | 1 |  |  |  |  |  |  |  |  |  |  |  |  |  | 4.774695 | 4.124217 |  |
| 17 | 15 | 8 | 1 | 1 |  |  |  |  |  |  |  |  |  |  |  |  | 5.414132 | 4.644205 |  |
| 18 | 41 | 19 | 9 | 2 | 1 | 1 |  |  |  |  |  |  |  |  |  |  | 6.087658 | 5.169079 |  |
| 19 | 153 | 55 | 18 | 7 | 3 |  |  |  |  |  |  |  |  |  |  |  | 6.805692 | 5.740846 |  |
| 20 | 385 | 170 | 60 | 21 | 16 | 4 | 1 |  |  |  |  |  |  |  |  |  | 7.561815 | 6.321243 |  |
| 21 | 1033 | 471 | 196 | 78 | 28 | 13 | 2 |  | 2 |  |  |  |  |  |  |  | 8.360546 | 6.899465 |  |
| 22 | 2376 | 1169 | 554 | 247 | 95 | 47 | 25 | 9 | 3 | 4 |  |  |  |  |  |  | 9.195005 | 7.534484 |  |
| 23 | 5289 | 2545 | 1334 | 611 | 297 | 128 | 45 | 21 | 8 | 2 | 3 | 1 | 1 |  |  |  | 10.06884 | 8.163966 |  |
| 24 | 10490 | 5900 | 3107 | 1525 | 732 | 366 | 187 | 59 | 35 | 13 | 4 | 4 | 1 | 1 |  |  | 10.98336 | 8.841776 |  |
| 25 | 19795 | 11435 | 6440 | 3459 | 1719 | 882 | 436 | 203 | 91 | 37 | 19 | 11 | 2 | 2 | 1 | 1 | 11.93981 | 9.494602 |  |
